# Rare histological prostate cancer subtypes: Cancer-specific and other-cause mortality

**DOI:** 10.1038/s41391-024-00866-4

**Published:** 2024-07-10

**Authors:** Carolin Siech, Mario de Angelis, Letizia Maria Ippolita Jannello, Francesco Di Bello, Natali Rodriguez Peñaranda, Jordan A. Goyal, Zhe Tian, Fred Saad, Shahrokh F. Shariat, Stefano Puliatti, Nicola Longo, Ottavio de Cobelli, Alberto Briganti, Benedikt Hoeh, Philipp Mandel, Luis A. Kluth, Felix K. H. Chun, Pierre I. Karakiewicz

**Affiliations:** 1 Cancer Prognostics and Health Outcomes Unit, Division of Urology, University of Montréal Health Center, Montréal, QC Canada; 2 Goethe University Frankfurt, University Hospital, Department of Urology, Frankfurt am Main, Germany; 3 Division of Experimental Oncology/Unit of Urology, URI, IRCCS Ospedale San Raffaele, Milan, Italy; 4 Vita-Salute San Raffaele University, Milan, Italy; 5 Department of Urology, IEO European Institute of Oncology, IRCCS, Milan, Italy; 6 Università degli Studi di Milano, Milan, Italy; 7 Department of Neuroscience, Science of Reproduction and Odontostomatology, University of Naples Federico II, Naples, Italy; 8 Department of Urology, AOU di Modena, University of Modena and Reggio Emilia, Modena, Italy; 9 Department of Urology, Comprehensive Cancer Center, Medical University of Vienna, Vienna, Austria; 10 Department of Urology, Weill Cornell Medical College, New York, NY USA; 11 Department of Urology, University of Texas Southwestern Medical Center, Dallas, TX USA; 12 Hourani Center for Applied Scientific Research, Al-Ahliyya Amman University, Amman, Jordan; 13 Department of Oncology and Haemato-Oncology, Università degli Studi di Milano, Milan, Italy

**Keywords:** Cancer epidemiology, Prostate cancer

## Abstract

**Background:**

To assess cancer-specific mortality (CSM) and other-cause mortality (OCM) rates in patients with rare histological prostate cancer subtypes.

**Methods:**

Using the Surveillance, Epidemiology, and End Results database (2004–2020), we applied smoothed cumulative incidence plots and competing risks regression (CRR) models.

**Results:**

Of 827,549 patients, 1510 (0.18%) harbored ductal, 952 (0.12%) neuroendocrine, 462 (0.06%) mucinous, and 95 (0.01%) signet ring cell carcinoma. In the localized stage, five-year CSM vs. OCM rates ranged from 2 vs. 10% in acinar and 3 vs. 8% in mucinous, to 55 vs. 19% in neuroendocrine carcinoma patients. In the locally advanced stage, five-year CSM vs. OCM rates ranged from 5 vs. 6% in acinar, to 14 vs. 16% in ductal, and to 71 vs. 15% in neuroendocrine carcinoma patients. In the metastatic stage, five-year CSM vs. OCM rates ranged from 49 vs. 15% in signet ring cell and 56 vs. 16% in mucinous, to 63 vs. 9% in ductal and 85 vs. 12% in neuroendocrine carcinoma. In multivariable CRR, localized neuroendocrine (HR 3.09), locally advanced neuroendocrine (HR 9.66), locally advanced ductal (HR 2.26), and finally metastatic neuroendocrine carcinoma patients (HR 3.57; all *p* < 0.001) exhibited higher CSM rates relative to acinar adenocarcinoma patients.

**Conclusions:**

Compared to acinar adenocarcinoma, patients with neuroendocrine carcinoma of all stages and locally advanced ductal carcinoma exhibit higher CSM rates. Conversely, CSM rates of mucinous and signet ring cell adenocarcinoma do not differ from those of acinar adenocarcinoma.

## Introduction

Acinar adenocarcinoma represents the dominant prostate cancer (PCa) histology [1–3]. Rare histological PCa subtypes include ductal, neuroendocrine, mucinous, or signet ring cell carcinoma [4–6]. Survival reports of prostate cancer patients with rare histological subtypes indicated less favorable oncological outcomes in most of these subtypes compared to acinar adenocarcinoma [1–3, 7–9]. However, previous analyses primarily addressed overall survival (OS) as study endpoints and did not distinguish between cancer-specific mortality (CSM) and other-cause mortality (OCM). Finally, no contemporary comprehensive analysis addressed CSM vs. OCM in a stage-specific fashion.

We addressed these knowledge gaps and postulated that CSM rates are higher than OCM rates in locally advanced and metastatic stages across all rare histological PCa subtypes, but not in localized stages. Moreover, we hypothesized that CSM rates in patients with some rare histological prostate cancer subtypes are higher than those of patients with the dominant histology, namely acinar adenocarcinoma. Finally, we also posited that these differences are less pronounced in patients with metastatic stages than in those with localized and locally advanced stages. To test these hypotheses, we relied on a contemporary population-based cohort of PCa patients within the Surveillance, Epidemiology, and End Results (SEER 2004–2020) database.

## Materials and methods

### Data source and study population

The SEER database (2004–2020; https://seer.cancer.gov/data/) provides cancer statistics covering approximately 47.9% of the United States population [10]. We focused on patients aged ≥18 years with histologically confirmed PCa (International Classification of Diseases [ICD-10] site code C61). Specifically, we identified the following rare histological subtypes: ductal (International Classification of Disease for Oncology [ICD-O-3] codes 8201/3, 8230/3, 8260/3, 8380/3, 8500/3, 8501/3, 8503/3, 8521/3, 8523/3, and 8552/3), neuroendocrine (ICD-O-3 codes 8013/3, 8020/3, 8021/3, 8041/3, 8042/3, 8044/3, 8045/3, 8240/3, 8244/3, and 8246/3, 8249/3), mucinous (ICD-O-3 codes 8480/3, and 8481/3), and signet ring cell carcinoma (ICD-O-3 codes 8490/3). Acinar adenocarcinoma patients (ICD-O-3 codes 8140/3, 8550/3, and 8551/3) were included as references. Other histological subtypes were excluded. Vital status or cause of death, and stage at presentation were known for all included patients. Autopsy- or death certificate-only cases were excluded. Based on the anonymously coded design of the SEER database, study-specific Institutional Review Board ethics approval was not required. The study has been conducted in accordance with the principles set in the Helsinki Declaration.

### Study endpoints

The primary study endpoint represented cancer-specific mortality (CSM), defined as all PCa-related deaths. The secondary endpoint consisted of other-cause mortality (OCM), defined as all deaths due to any cause except for mortality from PCa.

### Statistical analyses

First, baseline characteristics were tabulated. Descriptive statistics included frequencies and proportions for categorical variables as well as medians and interquartile ranges (IQR) for continuously coded variables. Second, age-adjusted annual incidence based on the year 2000 standard population of the United States was recorded. Third, smoothed cumulative incidence plots depicted the CSM and OCM of PCa patients. Subsequently, separate multivariable competing risks regression models were fitted to test for CSM differences between each histological prostate cancer subtype and acinar adenocarcinoma after accounting for OCM and adjusting for age at diagnosis (continuously coded) as well as local treatment (yes vs. no) for localized and locally advanced stage or chemotherapy (yes vs. no) for metastatic stage. All analyses were performed in a stage-specific fashion (localized vs. locally advanced vs. metastatic stage) within each histological PCa subtype (acinar vs. ductal vs. neuroendocrine vs. mucinous vs. signet ring cell carcinoma). Subgroup analyses included acinar adenocarcinoma with Gleason Grade Group 4 or 5 as reference. Statistical tests were two-sided with a level of significance set at *p* < 0.05. R software environment was used for statistical computing and graphics (R version 4.3.2; R Foundation for Statistical Computing, Vienna, Austria) [11].

## Results

### Descriptive characteristics and age-adjusted incidence

**Fig. 1:**
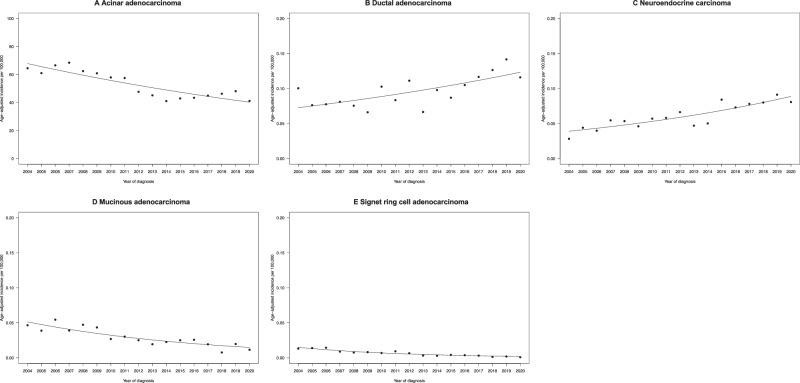
Age-adjusted incidence rates of newly diagnosed acinar adenocarcinoma and rare histological prostate cancer subtypes in the United States. Age adjustment has been performed according to the 2000 United States Standard Population.

Within the SEER database (2004–2020), we focused on 827,549 PCa patients. Of these, 824,530 (99.63%) harbored acinar, 1510 (0.18%) ductal, 952 (0.12%) neuroendocrine, 462 (0.06%) mucinous, and 95 (0.01%) signet ring cell carcinoma (Table 1). Median follow-up time was 78 (IQR 33–129) months in acinar, 45 (IQR 21–88) months in ductal, 8 (IQR 3–17) months in neuroendocrine, 96 (IQR 45–145) months in mucinous, and 24 (IQR 12–54) months in signet ring cell carcinoma patients. Patients with neuroendocrine carcinoma were oldest (median 70, IQR 63–79 years), and mucinous adenocarcinoma patients were youngest (median 63, IQR 57–69 years). Median PSA value was highest in signet ring cells (10.0, IQR 6.3–20.6 ng/ml) and lowest in neuroendocrine carcinoma patients (4.9, IQR 1.4–14.0 ng/ml). The metastatic stage was most prevalent in neuroendocrine (68%), followed by signet ring cell (15%), ductal (13%), mucinous (5%), and acinar carcinoma (5%). Local treatment rates were highest in mucinous (80%), signet ring cell (71%), and ductal adenocarcinoma  patients (68%). The chemotherapy rate was highest in neuroendocrine carcinoma patients (58%). Between 2004 and 2020, age-adjusted incidence increased from 0.10/100,000 person to 0.12/100,000 person for ductal and from 0.03/100,000 person to 0.08/100,000 person for neuroendocrine carcinoma (Fig. 1). Conversely, age-adjusted incidence decreased from 64.52/100,000 person in 2004 to 41.20/100,000 person in 2020 for acinar, from 0.05/100,000 person in 2004 to 0.01/100,000 person in 2020 for mucinous, and from 0.01/100,000 person in 2004 to 0.001/100,000 person in 2020 for signet ring cell adenocarcinoma.

**Table 1:** Descriptive characteristics of newly diagnosed patients with acinar adenocarcinoma and rare histological prostate cancer subtypes within the Surveillance, Epidemiology, and End Results database (2004-2020).

Characteristic	Acinar adenocarcinoma, *n* = 824,530 (99.63%)	Ductal adenocarcinoma, *n* = 1510 (0.18%)	Neuroendocrine carcinoma, *n* = 952 (0.12%)	Mucinous adenocarcinoma, *n* = 462 (0.06%)	Signet ring cell adenocarcinoma, *n* = 95 (0.01%)
**Age (in years)**^**a**^	66 (60, 73)	69 (62, 76)	70 (63, 79)	63 (57, 69)	67 (62, 72)
**Race/ethnicity**^**b**^					
Caucasians	572,360 (69%)	1072 (71%)	704 (74%)	325 (70%)	60 (63%)
African Americans	77,428 (9%)	145 (9%)	100 (11%)	62 (13%)	9 (10%)
Hispanics	117,806 (14%)	163 (11%)	89 (9%)	48 (11%)	18 (19%)
Others	56,936 (7%)	130 (9%)	59 (6%)	27 (6%)	8 (8%)
**PSA (in ng/ml)**^**a**^	6.6 (4.9, 10.3)	7.1 (4.6, 13.0)	4.9 (1.4, 14.0)	7.1 (5.2, 11.4)	10.0 (6.3, 20.6)
**Stage**^**b**^					
Localized	673,018 (82%)	789 (52%)	130 (14%)	321 (69%)	50 (53%)
Locally advanced	109,486 (13%)	522 (35%)	179 (19%)	119 (26%)	31 (33%)
Metastatic	42,026 (5%)	199 (13%)	643 (68%)	22 (5%)	14 (15%)

^a^Median (interquartile range).

^b^*n* (%).

### Cancer-specific and other-cause mortality in localized stage prostate cancer patients

**Fig. 2:**
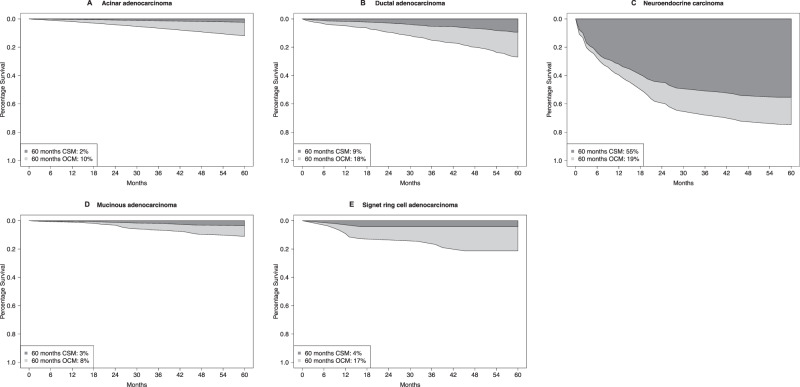
Smoothed cumulative incidence plots addressing cancer-specific mortality (CSM) and other-cause mortality (OCM) of patients with localized stage acinar adenocarcinoma or rare histological prostate cancer subtypes. CSM cancer-specific mortality, OCM other-cause mortality.

In localized stage, five-year CSM rates were 2% in acinar, 3% in mucinous, 4% in signet ring cells, 9% in ductal, and 55% in neuroendocrine carcinoma patients (Fig. 2A–E). Five-year OCM rates were 10% in acinar, 8% in mucinous, 17% in signet ring cell, 18% in ductal, and 19% in neuroendocrine carcinoma patients. Of all deaths recorded at five years of follow-up, cancer-specific deaths accounted for 17% in acinar, 27% in mucinous, 19% in signet ring cell, 33% in ductal, and 74% in neuroendocrine carcinoma. In multivariable competing risks regression models adjusting for age at diagnosis as well as local treatment and additional accounting for OCM, neuroendocrine carcinoma histology (HR 3.09, 95% CI 2.74–3.47; *p* < 0.001) independently predicted higher CSM rate relative to acinar adenocarcinoma (Table 2).

**Table 2:** Separate multivariable competing risks regression models addressing cancer-specific mortality (CSM) according to rare histological prostate cancer subtype vs. acinar adenocarcinoma in localized, locally advanced, and metastatic stage patients.

Stage	Histology	HR	(95% CI)	*p*-value
**Localized**	Ductal adenocarcinoma vs. Acinar adenocarcinoma^a^	1.07	(0.89, 1.28)	0.5
	Neuroendocrine carcinoma vs. Acinar adenocarcinoma^a^	**3.09**	(2.74, 3.47)	**<0.001**
	Mucinous adenocarcinoma vs. Acinar adenocarcinoma^a^	1.09	(0.64, 1.84)	0.8
	Signet ring cell adenocarcinoma vs. Acinar adenocarcinoma^a^	0.79	(0.33, 1.87)	0.6
**Locally advanced**	Ductal adenocarcinoma vs. Acinar adenocarcinoma^a^	**2.26**	(1.80, 2.85)	**<0.001**
	Neuroendocrine carcinoma vs. Acinar adenocarcinoma^a^	**9.66**	(6.81, 13.71)	**<0.001**
	Mucinous adenocarcinoma vs. Acinar adenocarcinoma^a^	1.05	(0.52, 2.12)	0.9
	Signet ring cell adenocarcinoma vs. Acinar adenocarcinoma^a^	2.08	(0.94, 4.61)	0.07
**Metastatic**	Ductal adenocarcinoma vs. Acinar adenocarcinoma^b^	1.14	(0.94, 1.37)	0.2
	Neuroendocrine carcinoma vs. Acinar adenocarcinoma^b^	**3.57**	(3.15, 4.05)	**<0.001**
	Mucinous adenocarcinoma vs. Acinar adenocarcinoma^b^	1.14	(0.67, 1.94)	0.6
	Signet ring cell adenocarcinoma vs. Acinar adenocarcinoma^b^	0.84	(0.35, 2.04)	0.7

^a^after adjusting for age at diagnosis, local treatment, and accounting for other-cause mortality.

^b^after adjusting for age at diagnosis, chemotherapy, and accounting for other-cause mortality.

CI confidence interval, HR hazard ratio, vs versus.

Bold values represent clinically meaningful and statistically significant odds ratios and *p* values.

### Cancer-specific and other-cause mortality in locally advanced stage prostate cancer patients

**Fig. 3:**
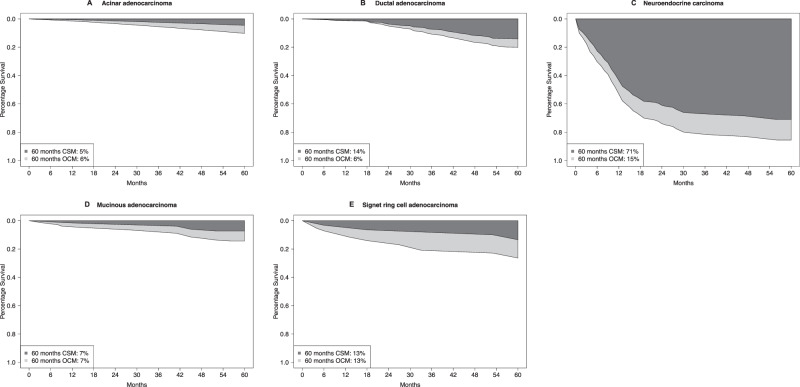
Smoothed cumulative incidence plots addressing cancer-specific mortality (CSM) and other-cause mortality (OCM) of patients with locally advanced stage acinar adenocarcinoma or rare histological prostate cancer subtypes. CSM cancer-specific mortality, OCM other-cause mortality.

In locally advanced stage, five-year CSM rates were 5% in acinar, 7% in mucinous, 13% in signet ring cell, 14% in ductal, and 71% in neuroendocrine carcinoma patients (Fig. 3A–E). Five-year OCM rates were 6% in acinar, 7% in mucinous, 13% in signet ring cell, 6% in ductal, and 15% in neuroendocrine carcinoma patients. Of all deaths recorded at five years of follow-up, cancer-specific deaths accounted for 46% in acinar, 50% in mucinous, 50% in signet ring cell, 70% in ductal, and 83% in neuroendocrine carcinoma. In multivariable competing risks regression models adjusting for age at diagnosis as well as local treatment and additional accounting for OCM, neuroendocrine (HR 9.66, 95% CI 6.81–13.71; *p* < 0.001) and ductal carcinoma histology (HR 2.26, 95% CI 1.80–2.85; *p* < 0.001) independently predicted higher CSM rate relative to acinar adenocarcinoma (Table 2).

### Cancer-specific and other-cause mortality in metastatic stage prostate cancer patients

**Fig. 4:**
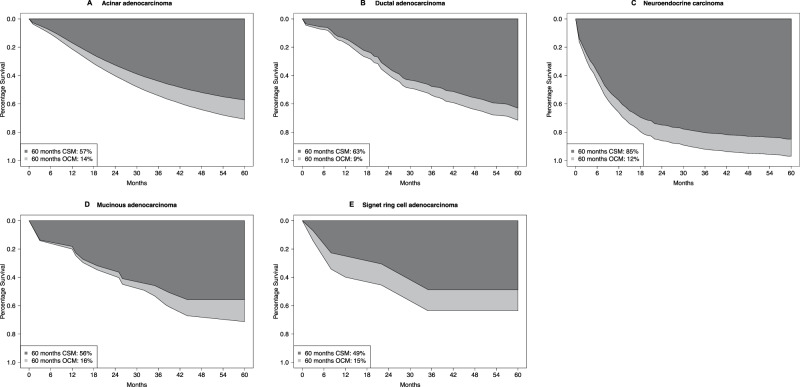
Smoothed cumulative incidence plots addressing cancer-specific mortality (CSM) and other-cause mortality (OCM) of patients with metastatic stage acinar adenocarcinoma or rare histological prostate cancer subtypes. CSM cancer-specific mortality, OCM other-cause mortality.

In metastatic stage, five-year CSM rates were 49% in signet ring cells, 56% in mucinous, 57% in acinar, 63% in ductal, and 85% in neuroendocrine carcinoma patients (Fig. 4A–E). Five-year OCM rates were 15% in signet ring cell, 16% in mucinous, 14% in acinar, 9% in ductal, and 12% in neuroendocrine carcinoma patients. Of all deaths recorded at five years of follow-up, cancer-specific deaths accounted for 77% in signet ring cell, 78% in mucinous, 80% in acinar, and 88% in ductal and neuroendocrine carcinoma. After adjustment for age at diagnosis as well as chemotherapy and additional accounting for OCM in multivariable competing risks regression models, neuroendocrine carcinoma histology (HR 3.57, 95% CI 3.15–4.05; *p* < 0.001) independently predicted higher CSM rate relative to acinar adenocarcinoma (Table 2).

### Subgroup analyses: cancer-specific mortality rates of rare histological prostate cancer subtypes relative to acinar adenocarcinoma with Gleason Grade Group 4 or 5

In subgroup analyses, multivariable competing risks regression models addressed CSM rates of rare histological prostate cancer subtypes relative to those of acinar adenocarcinoma with Gleason Grade Group 4 or 5 after accounting for OCM. In localized stage, neuroendocrine carcinoma histology independently predicted higher CSM rate (HR 6.27, 95% CI 4.42–8.88; *p* < 0.001), while mucinous adenocarcinoma histology independently predicted lower CSM rate (HR 0.49, 95% 0.31, 0.79; *p* < 0.001). No statistically significant differences in CSM rates were observed for signet ring cell (HR 0.88 95% 0.32, 1.89; *p* = 0.6) and ductal adenocarcinoma histology (HR 1.08, 95% CI 0.88–1.33; *p* = 0.5). In locally advanced stage, neuroendocrine (HR 8.25, 95% CI 5.99–11.36; *p* < 0.001) and ductal carcinoma histology (HR 1.40, 95% CI 1.12–1.74; *p* < 0.001) independently predicted higher CSM rates. Conversely, no statistically significant differences in CSM rates were observed for mucinous (HR 0.58 95% 0.29, 1.19; *p* = 0.1) and signet ring cell adenocarcinoma histology (HR 1.26 95% 0.59, 2.69; *p* = 0.6). In metastatic stage, neuroendocrine carcinoma histology independently predicted higher CSM rates (HR 2.97, 95% CI 2.63–3.34; *p* < 0.001). No statistically significant differences in CSM rates were observed for ductal (HR 1.07, 95% CI 0.89–1.28; *p* = 0.5), mucinous (HR 1.07, 95% CI 0.63, 1.82; *p* = 0.8), and signet ring cell adenocarcinoma histology (HR 0.78 95% 0.33, 1.85; *p* = 0.6).

## Discussion

In patients with rare histological PCa subtypes, it is unknown what proportion of deaths may be attributable to PCa (CSM) and what proportion of deaths is unrelated to PCa (OCM). We addressed this knowledge gap and made several noteworthy observations.

First, histological PCa subtypes represent a rare entity [1–5]. Within the current study (SEER 2004-2020), we identified 1510 ductal, 952 neuroendocrine, 462 mucinous, and 95 signet ring cell carcinoma patients among 827,549 PCa patients. This study cohort represents the most contemporary and second-largest cohort of patients with rare histological PCa subtypes. The largest cohort of patients with histological PCa subtypes was reported by Bronkema et al. who identified 1958 patients with ductal, 1304 with neuroendocrine, 1020 with mucinous, and 200 with signet ring cell carcinoma among 1,243,806 PCa patients within the National Cancer Database (NCDB 2004-2015) [3]. However, this study could only address OS. In consequence, the interplay between CSM and OCM that was addressed in the current study could not be reported. Other single- or multi-institutional studies relied on substantially smaller sample sizes [12–18]. Due to the rarity of histological PCa subtypes and small numbers of events, these PCa patients should ideally be investigated in population-based registries when cancer control outcomes are of interest.

Second, we identified important differences in baseline characteristics between patients with rare histological PCa subtypes and acinar adenocarcinoma. Within our cohort, patients with neuroendocrine carcinoma were the oldest (median 70) and mucinous adenocarcinoma patients were the youngest (median 63). In addition, the metastatic stage was most prevalent in neuroendocrine carcinoma (68%), and least prevalent in mucinous (5%) and acinar adenocarcinoma patients (5%). Similar observations were reported by Bronkema et al. (NCDB 2004-2015) and Marcus et al. (SEER 1973-2008) [1, 3]. However, the Bronkema et al. and Marcus et al. analyses only addressed OS as a study endpoint and did not distinguish between CSM or OCM. Due to the observed differences in age and stage distribution according to histological PCa subtype, it is essential to rely on stage-specific analyses as well as on additional adjustment for age in multivariable models when survival outcomes represent the endpoint of interest, as was done in the present study.

Third, we relied on smoothed cumulative incidence plots to quantify CSM and OCM rates at five years of follow-up. In localized stage, the proportion of PCa-specific deaths at five years of follow-up ranged from 74% in neuroendocrine to 33% in ductal, 27% in mucinous, 19% in signet ring cell, and 17% in acinar carcinoma. In locally advanced stage, PCa-specific deaths accounted for 83% in neuroendocrine carcinoma, followed by 70% in ductal, 50% in mucinous and signet ring cell, and 46% in acinar adenocarcinoma at five years of follow-up. Finally, in metastatic stage, PCa-specific deaths accounted for 88% in neuroendocrine carcinoma and ductal, 80% in acinar, 78% in mucinous, and 77% in signet ring cell adenocarcinoma at five years of follow-up. It is noteworthy that in neuroendocrine carcinoma of all stages, the majority of deaths represent PCa-specific events. In contrast, OCM plays a minor role in neuroendocrine carcinoma patients, especially in metastatic stage. Conversely, in localized stage acinar, signet ring cell, ductal, and mucinous as well as locally advanced stage acinar adenocarcinoma, the majority of deaths are attributable to other causes than PCa. In consequence, CSM plays a minor role in such patients. The currently reported observations do not only validate our hypothesis that CSM rates are higher than OCM rates in patients with locally advanced and metastatic stages across all rare histological PCa subtypes. The current observations also further validate the notion that CSM represents an essential survival endpoint. In consequence, lack of specific CSM consideration may result in misinterpretation of cancer control outcomes [1, 3, 8]. Finally, the above findings also validate the requirement of stage-specific stratifications.

Fourth, we compared CSM rates in patients with rare histological prostate cancer subtypes to those of patients with the dominant PCa histology, namely acinar adenocarcinoma. In multivariable competing risks regression analyses that adjust for age at diagnosis as well as respective treatment choice and additional account for OCM, localized (HR 3.09), locally advanced (HR 9.66), and metastatic neuroendocrine (HR 3.57), as well as locally advanced ductal carcinoma patients (HR 2.26) exhibited higher CSM rates relative to similar acinar adenocarcinoma patients. The current results cannot be directly compared to any previous study. Unfortunately, previous studies either only addressed overall mortality (OM) without stratifying between CSM and OCM [1, 3] or when CSM represented an endpoint of interest, the potential confounding effect of OCM was not addressed since competing risks regression models were not applied [2, 7, 9].

The main findings of the current study indicate substantial differences in CSM rates between histological PCa subtypes. Specifically, men with newly diagnosed neuroendocrine carcinoma experience less favorable cancer-specific survival regardless of stage. Moreover, these observations validate our hypothesis that CSM differences according to histological PCa subtype are less pronounced in patients with metastatic stages than in those with localized and locally advanced stages. Similarly, we recorded pronounced differences in CSM rates in locally advanced ductal vs. acinar adenocarcinoma patients, but not in localized and metastatic stages of these two subtypes. These observations disagreed with the findings of Knipper et al. [7], who did not account for the potentially confounding effect of OCM in multivariable models addressing CSM. Finally, patients with other rare histological PCa subtypes, such as mucinous and signet ring cell adenocarcinoma experience comparable CSM rates to acinar adenocarcinoma patients.

Taken together, our analyses quantifying CSM and OCM rates in patients with rare histological PCa subtypes relative to their acinar adenocarcinoma counterparts are important for clinical decision making. The consideration of OCM along with CSM may help clinicians caring for patients with rare histological PCa subtypes to better predict the treated natural history. Finally, the current results are also of great epidemiological value since they provide the most contemporary estimate of rare histological PCa subtype stage distribution and cancer control outcomes.

Despite its novelty, the study is not devoid of limitations. First, the present study relies on an observational and retrospective study design. This limitation applies to all previous studies that used large-scale databases, such as the NCDB [3, 8, 19, 20], or the SEER database [1, 2, 7, 21, 22]. Nonetheless, both the NCDB and SEER databases offer valuable opportunities to investigate rare histological subtypes and draw robust statistical conclusions. Second, despite the extensive scope of the SEER database, the number of patients with histological PCa subtypes is limited due to the rarity of ductal, neuroendocrine, mucinous, and signet ring cell carcinoma of the prostate. Third, ICD-O-3 histology codes are extracted from patient records and lack validation through central pathological review. However, the intrinsic biases linked with this approach are applicable to all histological subtypes. Fourth, pathological reporting of prostate cancer has evolved over time [5, 6]. In consequence, temporal trends of age-adjusted incidence rates may have been influenced by greater efforts to identify specific histological subtypes, such as ductal and neuroendocrine carcinoma, by the pathology community in recent years. Fifth, the present study relied on the SEER mortality code that is based on the cause of death certificates to distinguish between CSM and OCM. Penson et al. have confirmed the reliability of death certificates among prostate cancer patients [23]. Nevertheless, misclassification of the actual cause of death cannot be excluded. However, these intrinsic biases apply to all histological subtypes as well as to all previous analyses that used SEER mortality code [7, 21, 24]. Finally, the SEER database does not include earlier cancer-control endpoints than CSM and OCM. In consequence, other study endpoints that could be equally as interesting as CSM, such as biochemical recurrence or metastasis could not be addressed within the present database.

## Conclusions

Compared to acinar adenocarcinoma, patients with neuroendocrine carcinoma of all stages and locally advanced ductal carcinoma exhibit higher CSM rates. Conversely, CSM rates of mucinous and signet ring cell adenocarcinoma do not differ from those of acinar adenocarcinoma.

## Data Availability

All data generated or analyzed during this study are included in this published article.
